# Investigating Multidimensional Interoceptive Awareness in a Japanese Population: Validation of the Japanese MAIA-J

**DOI:** 10.3389/fpsyg.2018.01855

**Published:** 2018-10-08

**Authors:** Masayasu Shoji, Wolf E. Mehling, Martin Hautzinger, Beate M. Herbert

**Affiliations:** ^1^Clinical Psychology and Psychotherapy, University of Tuebingen, Tübingen, Germany; ^2^Department of Psychosomatic Medicine, National Center for Global Health Medicine, Kohnodai Hospital, Shinjuku, Japan; ^3^Department of Family and Community Medicine, Osher Center for Integrative Medicine, University of California, San Francisco, San Francisco, CA, United States

**Keywords:** bodily awareness, interoception, Japanese MAIA, cross-cultural bodily awareness, validation study

## Abstract

The Multidimensional Assessment of Interoceptive Awareness (MAIA) is a self-report instrument to assess relevant dimensions of bodily awareness. The aim of this study was to offer a Japanese version and adaptation of the MAIA (MAIA-J), as well as to analyse its psychometric properties in a Japanese population. The English MAIA was systematically forward and backward translated by bi-lingual Japanese experts; additionally, content validity aspects regarding language were discussed by a panel of experts. The MAIA-J was administered to 390 Japanese young adults (age: 20.3 ± 2.2), 67.7% women and 32.2% men. Exploratory factor analysis (EFA) reduced the questionnaire from 32 to 25 items and from 8 to 6 factors (Noticing, Not-Distracting, Attention Regulation, Emotional Awareness, Body Listening, and Trusting). The Japanese version showed appropriate indicators of construct validity and reliability, with Cronbach's α between 0.67 and 0.87 for the 6 MAIA-J dimensions. The findings demonstrate that MAIA-J has a slightly different factor structure compared to the original English MAIA. Results are discussed with respect to cultural differences. However, the study results support acceptable reliability of the MAIA-J in the Japanese sample, warranting its use for future studies with Japanese populations.

## Introduction

This study has been performed to systematically translate the Multidimensional Assessment of Interoceptive Awareness (MAIA) into Japanese language and to investigate the psychometric properties of the Japanese version of the MAIA (MAIA-J). The principal aim of this validation study was to apply MAIA-J in Japanese samples and to offer a Japanese MAIA version that allows to systematically investigate self-reported multidimensional aspects of interoceptive bodily awareness in future cross-cultural studies with Japanese participants.

Interoception refers to mechanisms how the brain senses and integrates signals from inside the body, providing a continuous mapping of the body's internal state. The perception of these bodily signals is associated to bodily states, such as thirst, hunger, pain, as well as stress (Pollatos et al., 2007; Craig, 2009; Herbert et al., 2010, 2011, 2012a; Herbert and Pollatos, 2012; Durlik et al., 2014), and has been shown to be closely associated with emotions, feelings and the perception of disease specific symptoms (Herbert et al., 2007a, 2011; Herbert and Pollatos, 2012; Terasawa et al., 2013; Khalsa and Lapidus, 2016; Pollatos et al., 2016; Khalsa et al., 2018). Individuals with more precise perception of interoceptive signals have been demonstrated to experience and process emotions more intensely, as well as to regulate emotions and behavior more adaptively (Herbert et al., 2007b, 2013; Herbert and Pollatos, 2012; Farb et al., 2013; Füstös et al., 2013; Tsakiris, 2017). The importance of interoception for adaptive and maladaptive behavior, as well as for psychopathology has gained growing interest, and dysfunctional interoception has been recognized to represent a core impairment across psychosomatic and psychiatric disorders (Herbert and Pollatos, 2012; Herbert et al., 2012b; Khalsa and Lapidus, 2016; Murphy et al., 2017). Interoceptive processes have also been recognized to represent a basic fundament for shaping the bodily self (Ainley et al., 2016; Tsakiris, 2017). Interoception has been demonstrated to represent a multidimensional construct differentiating interoceptive perception accuracy, as measured by objective behavioral tests (e.g., heartbeat perception, gastric perception, respiratory perception) (e.g., Schandry, 1981; Herbert et al., 2007a; Herbert and Pollatos, 2012; Pollatos et al., 2016; van Dyck et al., 2016), and subjective assessments of interoceptive sensations, as usually measured by self-reported measures, as well as a dimension of confidence-accuracy correspondence commonly labeled metacognitive awareness of interoceptive accuracy (Garfinkel et al., 2015). The subjective self-reported dimension of interoception depends on cognitive processes such as bodily awareness and associated evaluations, memories and attitudes (Mehling et al., 2009; Mehling, 2016), and its differential measurement is of outstanding importance.

To reliably capture different facets and potential changes of multiple dimensions of subjectively reported interoceptive bodily awareness that individuals may experience with mind-body interventions (Mehling et al., 2012), the MAIA questionnaire was developed through a systematic mixed-methods process. The resulting 32-item multidimensional instrument includes eight scales that separately assess multiple dimensions for the awareness of sensations, the quality of one's attention, the attitude toward and behavioral reaction to bodily sensations, and the individual style and capacity for mind-body integration.

The MAIA's convergent and divergent validity has been confirmed using different reliable and valid measures of constructs closely related to bodily awareness and mindful attention (Mehling et al., 2012). Up to today, the MAIA has been translated into 22 languages, including eight validation studies. Studies using adaptations of the MAIA in different languages for European and South-American cultures to a great degree confirmed its original factor structure, only with very few exceptions regarding item loadings (e.g., Bornemann et al., 2015; Brytek-Matera and Koziel, 2015; Calì et al., 2015; Valenzuela-Moguillansky and Reyes-Reyes, 2015; Grabauskaitė et al., 2017).

Up to now, there have been two studies that aimed at validating adaptations of MAIA in Asian countries (Chinese and Korean MAIA versions). Lin et al. (2017) offered a Chinese MAIA version and investigated Chinese populations. The authors reported confirmation of the original MAIA factor structure as well as acceptable reliability and validity in psychometric testing. The Korean version and pilot validation study by Wan-Suk et al. (2016) was published in Korean language, and reported results of the exploratory factor analysis (EFT) demonstrate that the Korean MAIA shows a different factor structure with 32 items and 6 factors (Noticing, Accept, Attention Regulation, Mind-Body Connection Awareness, Return to Body and Trusting). The Korean MAIA includes three factors with a different structure of item-factor loadings that have been re-named by the authors compared to the original English MAIA version. This version has been reported to show sufficient reliability and validity in Korean healthy young adult samples.

Given these results and prior cross-cultural studies on subjective awareness of the body, the emphasis on body processes and interoceptive accuracy (heartbeat perception accuracy) between “Western” and “Asian” cultures (see for an overview Ma-Kellams et al., 2012; Maister and Tsakiris, 2014; Ma-Kellams, 2014) it may not be surprising to find differences in factor structure of self-reported subjective measures of interoceptive bodily awareness as measured by the MAIA in Japanese populations. This could potentially be understood in a prominent conceptual view that Western and Eastern-Asian cultural differences are associated with differences in self-perception (Markus and Kitayama, 1991). In this view Western individuals tend to focus more on “independent” self-construal, where they think of the self as unique and distinct from social context, as well as value individuality. Conversely, East Asian cultures tend to emphasize an “interdependent” self-construal, in which interpersonal relationships are stressed. Here, the self is considered as embedded in a social context, and group harmony and cooperation are of relevance (e.g., Heine, 2001). These differences in emphasis of self-construal may be associated with differences in information-processing biases in East Asian and Western cultures. Accordingly, East Asian individuals tend to focus on a holistic processing style, and Western individuals tend toward a more analytical information-processing style (Kühnen et al., 2001). Additionally, cultural differences of self-construal have also been highlighted to be reflected in differences in language (Tung, 1994), and language also stands as a key mechanism for transmitting cultural ideas (Gudykunst et al., 1996; Tomasello, 1999). These factors also shape cross-cultural variation in the degree to which individuals emphasize bodily experiences and vice versa (Ma-Kellams, 2014). Therefore, it may be expected that the factor structure of a Japanese version of the MAIA as examined in a native Japanese population of young adults may be different compared to the original Western English MAIA version.

This article presents the translation and Japanese adaptation of the MAIA (MAIA-J), as well as the evaluation of its psychometric properties in a Japanese population.

## Materials and methods

### Participants

The sample of the present study consisted of 390 young adults and includes students and healthy young adults of Tokyo Kasei University, Sapporo international University, Tukuba International University and Kansei University in Japan, 126 men and 264 women with a mean age of 20.3 years (SD = 2.2) (Table 1). All participants were healthy native Japanese living in Japan without psychiatric and somatic diseases and without substance abuse (by self-report) and gave written informed consent. The study was conducted in accordance with the Declaration of Helsinki.

**Table 1:** Number of subjects and mean age for EFA and CFA.

	**Number of subjects**	**Mean (±SD) age**
Total	390	20.27 ± 2.21
Male	126 (32.2%)	20.57 ± 2.21
Female	264 (67.7%)	20.22 ± 2.21

For exploratory (EFA) and confirmatory factor analyses (CFA) the full sample was included without missing data. Due to missing data for additional appropriate self-report scales, construct validity of the MAIA-J was examined in *N* = 251 (60 men and 191 women; mean age = 20.1 ± 2.4) (Table 2).

**Table 2:** Number of subjects and mean of age for analyses of construct validity.

	**Number of subjects**	**Mean age (±SD)**
Total	251	20.06 ± 2.40
Male	60 (23.9%)	19.75 ± 1.80
Female	191 (76.1%)	20.16 ± 2.55

### Procedure and translation of MAIA

This study is a cross-sectional study in which participants were asked to complete questionnaires, including the Japanese translation of the English MAIA (Mehling et al., 2012). Students attending psychology classes at Tokyo Kasei University, Sapporo International University and Tukuba International University were invited to participate. Permissions to conduct the survey were obtained from the Universities.

Similar to the Chinese version of the MAIA (Lin et al., 2017), a systematic forward-backward translation of the English MAIA into Japanese was accomplished by considering a decentered translation strategy in order to retain equivalent meaning across languages and cultures (Beaton et al., 2000).

Translations were produced by an independent translation office, as well as by six native Japanese experts (Psychology students and experts from Psychology, including author MS). Three of them used to live in the UK and two in the USA. Additionally, a panel of native speaking Japanese with English language expertise examined the content validity of the translation. Finally, comments made during every step of this translation process were discussed in an international expert panel including MS, BMH, WM and SO (see Acknowledgment). This expert panel discussed and qualitatively evaluated all items for potential difficulties of understanding and differences in meaning regarding language and terminology. In this way translation problems were thoroughly considered by experts for both language and psychology. The resulting field-tested Japanese version preserved all items and the structure of the original questionnaire (see Appendix).

Additionally, MS conducted short cognitive interviews with all Japanese participants that completed the MAIA. Interviewees were asked whether they had questions or comments regarding the wording or understanding of the MAIA items. This was done in order to get some qualitative insight into potential language-related problems with the Japanese translation or with conceptual issues of MAIA terms that informed the discussion of the psychometrics of the MAIA-J.

### Instruments

In accordance with the original MAIA study (Mehling et al., 2012), the following questionnaires were used in order to test the psychometric properties of the MAIA-J with its construct validity (convergent and discriminant) in the Japanese sample. To allow for comparison with prior results, only standardized questionnaires were used that had also been applied in the original MAIA publication (Mehling et al., 2012).

#### Multidimensional assessment of interoceptive awareness (MAIA)

The MAIA was based on a conceptual delineation of multiple interoceptive perception processes and represents a 32-items self-report questionnaire with eight scales (Mehling et al., 2012): The scales are: (1) Noticing (awareness of uncomfortable, comfortable, and neutral body sensations), (2) Not-Distracting (tendency not to ignore or distract oneself from sensations of pain or discomfort), (3) Not-Worrying (tendency not to worry or experience emotional distress with sensations of pain or discomfort), (4) Attention Regulation (ability to sustain and control attention to body sensations), (5) Emotional Awareness (awareness of the connection between body sensations and emotional states), (6) Self-Regulation (ability to regulate distress by attention to body sensations), (7) Body-Listening (active listening to the body for insight), and (8) Trusting (experience of one's body as safe and trustworthy). The MAIA has been developed in individuals experienced in mind-body practices and validated in primary care patients with past or current low back pain that had no prior experience with body-mind therapies (Mehling et al., 2012, 2013). Items are answered on a 6-point Likert scale (0 to 5) with higher scores indicating higher interoceptive bodily awareness. As two of its scales (Not-Distracting and Not-Worrying) with only three items each have shown acceptable but weak reliability for internal consistency with Cronbach's alphas of 0.66 and 0.67 (Mehling et al., 2012), an improved Version 2 of the MAIA is underway but was not yet available for the current study. Cronbach's alphas in the other 6 scales were between 0.74 and 0.90 (Mehling et al., 2012).

#### Five facet mindfulness questionnaire (FFMQ)

The FFMQ (Baer et al., 2006; Sugiura et al., 2012) was chosen as a measure of mindful attention and bodily awareness. The FFMQ is a 39-item, multidimensional self-report scale, and one of the most widely used and established measures for mindfulness. It includes 5 subscales: Observing (F1, OBS: ability to observe body sensations among various other stimuli), Describing (F2, DSC: describing emotions), Acting with Awareness (F3, AWA: attending to one's activities of the moment), Nonjudging (F4, NOJ: attend to body sensations), and Nonreactivity (F5, NOJ: accepting body sensations). Items are rated on 5-point Likert scales from 1 (never or very rarely true) to 5 (very often or always true). FFMQ subscale internal-consistency reliabilities ranged from 0.76 to 0.92 (Baer et al., 2008). Japanese reliabilities were in an equivalent range (Sugiura et al., 2012).

#### Difficulties in emotion regulation scale (DERS)

The DERS was selected as a measure for the ability to regulate emotions. Interoception has been demonstrated to be relevant for emotion regulation (Füstös et al., 2013), however, attendance to the body is not explicitly a measure of emotion regulation. To examine the relationship between emotion regulation and the bodily awareness scales of the MAIA, it is of interest that the MAIA includes a scale “Emotional Awareness.”

The original DERS is a 36 items self-report questionnaire (Gratz and Roemer, 2004). Items are scored on five-point Likert scales from 1 (almost never) to 5 (almost always) and consists of 6 dimensions: Nonacceptance of emotional responses (NAC), Difficulties in engaging in goal-directed behaviors (GLS), Impulse control difficulties (IMP), Lack of emotional awareness (AWR), Limited access to emotion regulation strategies (STR), and Lack of emotion clarity (CLR). Internal consistencies for the six subscales ranged from 0.80 to 0.90. Yamada and Sugie (Yamada and Sugie, 2012) developed a Japanese version of the DERS (J-DERS) that was validated as a scale with 16 items and 4 factors. Its factors F1–F4 are most equivalent to NAC, IMP, STR, and AWR (F1: difficulties in acceptance of emotions, F2: difficulties in behavior control, F3: dysfunctions in emotion regulation strategies, F4: difficulties of emotional awareness). Internal consistencies are comparable to the original version.

#### State-trait anxiety inventory (STAI)

We used a Japanese version of the STAI-T trait anxiety (Spielberger, 1989) scale for divergent validity assessment of the MAIA scales. The STAI-T is a 20-item self-report questionnaire with items rated on a 4-point Likert scale from 1 (almost never) to 4 (almost always). This scale is an internationally widely used standard measure with excellent validity.

#### Pain catastrophizing scale (PCS)

A Japanese version of the PCS (Sullivan et al., 1998; Hirofumi and Yuji, 2007) was included as a measure of distress in response to bodily pain. The PCS is a 13-item self-report instrument to assess catastrophizing in response to pain sensations (Sullivan et al., 1995). This questionnaire consists of three subscales: Rumination, the inability to inhibit persisting pain-related thoughts (RUM), Helpless, identifying worry about pain and the sense of being overwhelmed by it, (HLP), and Magnification, the concern that the pain will get worse or have a negative outcome (MAG). Items are rated on 5-point Likert scales from 0 (not at all) to 4 (all the time). It has been demonstrated to have excellent validity (Hirofumi and Yuji, 2007).

### Statistical analyses

In order to determine the factor structure of the Japanese MAIA items and whether the factor structure of the original version would replicate in the Japanese version, we conducted an exploratory factor analysis (EFA) with maximum likelihood estimation and varimax rotation (extraction criterion: eigenvalue > 1).

Cronbach's alpha coefficient and corrected item scale correlations were used to assess the internal consistency reliability of the scales. Convergent and discriminant validity of MAIA-J were assessed by calculation Pearson intercorrelations for MAIA, FFMQ, DERS, PCS, and STAI-T. Regarding pre-test hypotheses for correlations between these scales and subscales, we followed the same hypotheses as described in extensive detail in the original study (Mehling et al., 2012).

The full sample of *N* = 390 was available to derive Cronbach's alphas for internal consistency of the MAIA scales. For convergent and discriminant validity, a sample of *N* = 251 with complete data was available.

Statistical analyses were conducted using IBM SPSS Statistics for Windows 20.0. (IBM Inc., Armonk, NY, USA).

## Results

### Factor structure of the japanese maia (MAIA-J)

Exploratory Factor Analysis (EFA) results showed a different factor structure for the MAIA-J compared to the original English MAIA, German (Bornemann et al., 2015), Italian (Calì et al., 2015) or Spanish (Valenzuela-Moguillansky and Reyes-Reyes, 2015) MAIA versions, that all demonstrated equivalent factor structures. Our EFA reduced the MAIA-J from 32 to 25 items, and its results demonstrated that a 6-factor model represented the best model fit (see Table 3).

**Table 3:** Exploratory Factor analysis of the MAIA-J (Items and standardized EFA loadings).

**MAIA-J**		**Standardized**	**Item-scale**
**Items and factors**		**loadings**	**correlations**
**ATTENTION REGULATION: CRONBACH'S** α = **0.87**			
14	I can return awareness to my body if I am distracted.	0.79	0.83
15	I can refocus my attention from thinking to sensing my body.	0.76	0.83
13	When I am in conversation with someone, I can pay attention to my posture.	0.63	0.72
17	I am able to consciously focus on my body as a whole.	0.54	0.71
12	I can maintain awareness of my inner bodily sensations even when there is a lot going on around me.	0.53	0.74
16	I can maintain awareness of my whole body even when a part of me is in pain or discomfort.	0.52	0.71
11	I can pay attention to my breath without being distracted by things happening around me.	0.47	0.69
**BODY LISTENING: CRONBACH'S** α = **0.84**			
29	I listen to my body to inform me about what to do.	0.69	0.84
27	I listen for information from my body about my emotional state.	0.65	0.85
28	When I am upset, I take time to explore how my body feels.	0.63	0.84
26	When I am caught up in thoughts, I can calm my mind by focusing on my body/breathing.	0.51	0.76
**NOTICING: CRONBACH'S** α = **0.74**			
18	I notice how my body changes when I am angry.	0.69	0.75
19	When something is wrong in my life I can feel it in my body.	0.60	0.70
1	When I am tense I notice where the tension is located in my body.	0.52	0.75
2	I notice when I am uncomfortable in my body.	0.43	0.66
4	I notice changes in my breathing, such as whether it slows down or speeds up	0.41	0.65
**EMOTIONAL AWARENESS: CRONBACH'S** α = **0.85**			
22	I notice how my body changes when I feel happy / joyful.	0.71	0.84
21	I notice that my breathing becomes free and easy when I feel comfortable.	0.69	0.89
20	I notice that my body feels different after a peaceful experience.	0.59	0.84
**TRUSTING: CRONBACH'S** α = **0.83**			
31	I feel my body is a safe place.	0.78	0.89
30	I am at home in my body.	0.61	0.88
32	I trust my body sensations.	0.46	0.83
**NOT-DISTRACTING: CRONBACH'S** α = **0.67**			
5	I do not notice (I ignore) physical tension or discomfort until they become more severe.	0.72	0.82
6	I distract myself from sensations of discomfort.	0.71	0.80
7	When I feel pain or discomfort, I try to power through it.	0.48	0.70

Low factor loadings as well as item cross-loadings for the following seven items indicated the exclusion of these items from the MAIA-J. Factor loadings were < 0.43 for items 23: “When I feel overwhelmed I can find a calm place inside,” 24: “When I bring awareness to my body I feel a sense of calm,” 25: “I can use my breath to reduce tension” from the original “Self-Regulation” scale; and factor loadings were < 0.30 for items 3: “I notice where in my body I am comfortable,” 8: “When I feel physical pain, I become upset,” 9: “I start to worry that something is wrong if I feel any discomfort,” 10: “I can notice an unpleasant body sensation without worrying about it” from the original “Not-Worrying” scale. Accordingly, the original MAIA scales “Self-Regulation” and “Not-Worrying” were deleted from the MAIA-J. Cronbach's alpha coefficients of these scales were < 0.38.

The remaining 6 factors accounted for 53.33% of the total variance. Table 3 summarizes EFA results and demonstrates relevant changes in factor loadings of items and factor structure of MAIA-J. These can be summarized as follows:

#### Attention regulation (7 items)

The items in Factor 1 were identical to the items of the “Attention Regulation” scale. This factor contained all 7 items originally describing the ability to maintain and control attention to bodily sensations in the original MAIA (Mehling et al., 2012).

#### Body listening (4 items)

The “Body Listening” factor was described by all original items of this factor, and additionally included item 26: “When I am caught up in thoughts, I can calm my mind by focusing on my body/breathing.” This item originally loaded on the (in the MAIA-J non-existent) “Self-Regulation” factor.

#### Noticing (5 items)

The “Noticing” factor of the MAIA-J comprised 5 items: it included 3 items (item 1, 2, 4) of the original “Noticing” scale, as well as 2 additional items. These items (item 18: “I notice how my body changes when I am angry” and item 19: “When something is wrong in my life I can feel it in my body”) “shifted” from the original “Emotional Awareness” scale to the “Noticing” scale.

#### Emotional awareness (3 items)

The factor “Emotional Awareness” of MAIA-J comprises 3 items (items 20, 21, 22) from the original 5 items MAIA scale (items 18 and 19 “shifted” to the “Noticing” scale). “Emotional Awareness” contains items describing a connection of body sensing and emotional states.

#### Trusting (3 items)

The items of the MAIA-J Factor 5 did not differ from the original MAIA Trusting scale and included the 3 items describing an experience of one's body as safe and trustworthy.

#### Not-distracting (3 items)

Comparably, “Not-Distracting” scale included the 3 original items of the English MAIA describing the tendency not to ignore or distract oneself from bodily sensation of discomfort and pain.

### Reliability and scale-scale correlations

Table 4 shows mean values, standard deviation and internal consistencies of the MAIA-J as well as ranges of item-scale correlations. There were significant intercorrelations ranging from 0.65 to 0.91.

**Table 4:** Cronbach's alpha coefficients, range of inter-item-scale correlations and descriptive statistics (means ± SD) for MAIA-J.

**Scales**	**Cronbach's α**	**Range of item-scale correlations**	**Mean ±SD**
Attention regulation (7 items)	0.87	0.83–0.69	2.63 ± 0.86
Body listening (4 items)	0.84	0.85–0.76	2.26 ± 1.03
Noticing (5 items)	0.74	0.65–0.75	2.96 ± 0.87
Emotional awareness (3 items)	0.85	0.91–0.84	2.88 ± 1.12
Trusting (3 items)	0.83	0.89–0.83	2.67 ± 1.10
Not-distracting (3 items)	0.67	0.82–0.70	2.44 ± 0.95

Internal consistency reliability is demonstrated by Cronbach's alpha coefficients of the scales (Table 4). Cronbach's alphas of the 6 MAIA-J scales ranged between 0.67 and 0.87 and demonstrated appropriate to high internal consistency.

Pearson intercorrelations of the six MAIA-J scales are depicted in Table 5 and ranged from - 0.13 to 0.65. The highest correlations were between Body Listening and Trusting (0.65), Attention Regulation and Body listening (0.63), Attention Regulation and Trusting (0.61).

**Table 5:** Pearson intercorrelations among the 6 MAIA-J factors.

	**F1**		**F2**		**F3**		**F4**		**F5**		**F6**	
F1										
F2	0.63	^**^									
F3	0.52	^**^	0.48	^**^						
F4	0.49	^**^	0.58	^**^	0.58	^**^				
F5	0.61	^**^	0.65	^**^	0.46	^**^	0.54	^**^		
F6	−0.13	^*^	−0.03		0.02		0.07		0.05	

F1, Attention Regulation; F2, Body Listening; F3, Noticing; F4, Emotional Awareness; F5, Trusting; F6, Not-Distracting.

p < 0.05^*^

p < 0.01^**^.

In summary, these results indicate appropriate construct validity and reliability of the MAIA-J.

### Construct validity of MAIA-J

Convergent and discriminant validity was analyzed in 251 participants (see Table 2) by calculating Pearson correlations of adapted MAIA-J scales (6-factors) and scores of FFMQ, DERS, STAI-T, and PCS (Table 6).

**Table 6:** Pearson's correlations of the FFMQ, DERS, STAI-T, PCS, STSS and MAIA-J.

	**MAIA_F1**		**MAIA_F2**		**MAIA_F3**		**MAIA_F4**		**MAIA_F5**		**MAIA_F6**	
**MINDFUL AWARENESS**												
FFMQ_F1	0.41	^**^	0.39	^**^	0.41	^**^	0.46	^**^	0.43	^**^	−0.03	
FFMQ_F2	0.51	^**^	0.44	^**^	0.21	^**^	0.17	^**^	0.51	^**^	−0.06	
FFMQ_F3	−0.11		−0.07		−0.18	^**^	−0.15	^*^	0.01		0.24	^**^
FFMQ_F4	0.34	^**^	0.39	^**^	0.19	^**^	0.19	^**^	0.44	^**^	0.12	
FFMQ_F5	0.16	^*^	0.18	^**^	−0.03		−0.02		0.21	^**^	0.19	^**^
FFMQ_T	0.42	^**^	0.44	^**^	0.19	^**^	0.20	^**^	0.54	^**^	0.18	^**^
**MEASURE OF EMOTION REGULATION**												
DERS_F1	−0.14	^*^	−0.07		0.04		0.02		−0.20	^**^	−0.24	^**^
DERS_F2	−0.25	^**^	−0.24	^**^	0.05		−0.02		−0.26	^**^	−0.14	^*^
DERS_F3	−0.20	^**^	−0.18	^**^	0.11		0.02		−0.24	^**^	−0.15	^*^
DERS_F4	−0.32	^**^	−0.33	^**^	−0.17	^**^	−0.14	^*^	−0.40	^**^	−0.26	^**^
DERS_T	−0.27	^**^	−0.24	^**^	0.02		−0.03		−0.32	^**^	−0.24	^**^
**MEASURE OF ANXIETY**												
STAI_T	−0.29	^**^	−0.34	^**^	0.02		−0.09		−0.46	^**^	−0.23	^**^
PCS_F1	−0.06		−0.01		0.11		0.08		−0.03		−0.14	^*^
PCS_F2	−0.11		−0.06		0.06		−0.01		−0.14	^*^	−0.19	^**^
PCS_F3	0.00		0.09		0.16	^*^	0.05		0.05		−0.16	^*^
PCS_T	−0.08		−0.01		0.12		0.04		−0.06		−0.20	^**^

p < 0.05^*^, p < 0.01^**^.

MAIA, Multidimensional Assessment of Interoceptive Awareness; MAIA F1, Attention Regulation; MAIA F2, Body Listening; MAIA F3, Noticing; MAIA F4, Emotional Awareness; MAIA F5, Trusting; MAIA F6, Not-Distracting.

FFMQ, Five Facet Mindfulness Questionnaire; FFMQ F1, Observing; FFMQ F2, Describing; FFMQ F3, Acting with awareness; FFMQ F4, Nonjudging of inner experience; FFMQ F5, Nonreactivity to inner experience.

Difficulties in Emotion Regulation Scale, DERS; DERS F1, Difficulties in acceptance of emotions; DERS F2, Difficulties in behavior control; DERS F3, Dysfunctions in emotion regulation strategies; DERS F4, Difficulties of emotional awareness; DERS T, Total score.

*Pain Catastrophizing Scale, PCS; PCS F1, Rumination; PCS F2, Helpless; PCS F3, Magnification; PCS T, Total score*.

Table 6 summarizes that most 6 MAIA-J scales were significantly and positively correlated with scores of FFMQ subscales and total FFMQ score, nearly equivalent to the findings of the original English MAIA study (Mehling et al., 2012), confirming our pre-test hypotheses (the same as in Mehling et al. (2012) and the questionnaire's convergent validity.

In difference to the original English MAIA study “Acting with Awareness” (AWA) scale of FFMQ was weakly negative correlated with the MAIA-J “Noticing” scale (*r* = −0.18, *p* < 0.01) as well as with “Emotional Awareness” (*r* = −0.15, *p* < 0.05).

Furthermore, all MAIA-J scales showed overall negative or non-significant correlations with DERS scales (−0.24 to −0.32) and STAI trait anxiety (−0.23 to −0.49), suggesting that emotion regulation difficulties and trait anxiety are also negatively associated with bodily awareness measures of MAIA-J.

Correlations with STAI trait anxiety were slightly lower than in the English MAIA study, and there were no significant correlations between MAIA-J scales “Noticing” as well as “Emotional Awareness” and STAI trait anxiety. This is different to the significant and negative correlations of all original MAIA scales with STAI trait anxiety in the US sample of the English MAIA (Mehling et al., 2012).

PCS scales were also negatively or not correlated to MAIA-J scales (−0.34 to 0.09), comparable to the original MAIA, with the exception of PCS subscale “Magnification.” PCS “Magnification” scale was slightly positive correlated to MAIA-J scale “Noticing.” In the original MAIA study with US samples experienced in mind-body practices, all MAIA scales were clearly negatively correlated with PCS subscales (Mehling et al., 2012).

## Discussion

This study aimed to translate the MAIA into Japanese and to assess reliability and validity of the MAIA-J in a Japanese population. The results show that the MAIA-J has a slightly different factor structure compared to the original MAIA as assessed in US and European samples. Exploratory factor analysis demonstrated that MAIA-J consisted of six factors, for which we maintained the original labels, including: Attention Regulation, Body Listening, Noticing, Emotional Awareness, Trusting, and Not-Distracting.

Results also demonstrated good internal consistency of MAIA-J scales, except of “Not-Distracting” (alpha = 0.64). However, this is comparable to the original version of the MAIA, as well as to the German and Spanish MAIA versions that showed even lower Cronbach's α than MAIA-J results (English: 0.66, German: 0.56, and Chile-Spanish: 0.49). This may be explained by the small number of items of this scale that diminishes Cronbach's α scores. Importantly, seven items (3, 8, 9, 10, 23, 24, and 25) that were originally defining “Self-Regulation” and “Not Worrying” scales were excluded from the MAIA-J. As has been summarized in the introduction section the “Not-Worrying” factor is also a weaker factor regarding reliability for internal consistency in the original MAIA with lower Cronbach alpha of 0.67, and it comprises only 3 items.

Furthermore, qualitative analyses of notes and discussions of the translation expert panel, as well as of the cognitive interviews of the Japanese participants, suggest that this may be partially due to language and conceptual translation problems of some of these items into Japanese language. Particularly, translation problems were mentioned with items of the Not-Worrying scale: items 8 (“When I feel physical pain, I become upset”), 9 (“I start to worry that something is wrong if I feel any discomfort”), and 10 (“I can notice an unpleasant body sensation without worrying about it”) that are defining the “Not Worrying” scale.

The translation expert panel concluded that these items were difficult to translate into Japanese without changing its conceptual meaning. A major problem also emerged for the term “upset,” which could not be adequately translated into Japanese. The translated term was closer to the meaning of “anger” in Japanese language. The translation problem with “upset” has its parallel with a translation problem reported for the Spanish-Chilean MAIA version. Valenzuela-Moguillansky and Reyes-Reyes (2015) stated that “upset” when translated into Spanish does not really express “worrying” but “anger.”

Interestingly, the MAIA “Self-Regulation” scale, which originally was described as assessing the ability to regulate distress by attention to body sensations, also disappeared in the MAIA-J validation. One MAIA “Self-Regulation” item was maintained and shifted to “Body Listening” in the MAIA-J (item 26: “When I am caught up in thoughts, I can calm my mind by focusing on my body/breathing”). Importantly, the MAIA-J “Noticing” and “Emotional Awareness” scales were affected by differences in item loadings and changes in factor loadings (Table 3). The MAIA “Noticing” scale had been originally defined as assessing the awareness of uncomfortable, comfortable, or neutral body sensations. “Emotional Awareness” had been defined as assessing the ability to attribute specific physical sensations to physiological manifestations of emotions, which was viewed as representing an internal process involving more developed interoceptive awareness or meta-awareness that has matured beyond reflexive reactivity with fear and worry about unfamiliar or irritating bodily sensations (Mehling et al., 2012).

One possible interpretation for the changes in factor loadings for these three MAIA scale items (two Emotional Awareness MAIA items moved to the MAIA-J Noticing factor; both factors were correlated at 0.58) could be that “Noticing” and “Emotional Awareness” may be less “disentangled” in Japanese culture, implying less of a tendency to separate bodily sensations and emotional processes (Komaki et al., 2003). This would be in line with reports that East-Asians appear to demonstrate a greater emphasis on their bodily states when describing themselves and their emotional experiences as well as perceiving bodily and psychological states to be closely intertwined (Ma-Kellams, 2014).

Alternatively, our findings may also be interpreted in a way that bodily signals are more intensely emotionally evaluated, including a potentially more negative bias in evaluating bodily sensations, when compared to Western culture. In accordance with this, Japanese individuals (undergraduates) have shown lower body esteem ratings compared to Westerners or Chinese, potentially resulting from a higher general tendency for self-effacement, and social anxiety (Kowner, 2002). Similar findings, revealing lower self-esteem have also been reported for Korean women compared to US women (Jung and Lee, 2006).

Regarding convergent construct validity, most of the 6 MAIA-J scales were significantly and positively correlated with scores of FFMQ subscales and total FFMQ score, measuring mindfulness extending beyond bodily awareness to the awareness of exteroceptive stimuli and thoughts. This is nearly equivalent to the findings of the English MAIA study (Mehling et al., 2012), demonstrating convergent validity. It also shows that the MAIA is a measure of mindful bodily awareness rather than anxiety-driven bodily awareness (Mehling, 2016) and this has been also confirmed for the MAIA-J.

One relevant difference to original English MAIA study results is that the “Acting with Awareness” (AWA) scale of the FFMQ was slightly negatively correlated with MAIA-J “Noticing” scale (*r* = −0, 18, *p* < 0.01) as well as with “Emotional Awareness” subscale (*r* = −0.15, *p* < 0.05). FFMQ “Acting with Awareness” (AWA) describes attending to one's activities of the present moment and is not primarily addressing awareness of one's internal bodily sensations. Both MAIA scales were weakly but statistically significantly positively correlated with this FFMQ subscale in the Western MAIA study (Mehling et al., 2012). The original sample was from participants experienced with mind-body practices, for whom acting with awareness may be more strongly associated with their emotional awareness compared to individuals less experienced in mind-body practices.

These results for MAIA-J may be due to the fact that especially the MAIA-J “Noticing” and “Emotional Awareness” scales slightly changed their item structure compared to the original MAIA as described above. The MAIA-J “Noticing” scale also includes relevant emotionally connoted items from the original “Emotional Awareness” scale (see Table 3), suggesting greater overlap of emotional evaluation and awareness of bodily sensations. Furthermore, the difference in correlations regarding MAIA-J “Noticing” and “Emotional Awareness” with FFMQ-AWA may suggest that recognizing one's bodily reactivity and one's emotional awareness based on one's bodily signals or one's actions in the present moment may not be as “coherent” as in Western cultures.

This might be explained by potential cultural differences of self-construal. East Asian cultures tend to hold more “interdependent” self-construal, in which interpersonal relationships are stressed, and the self is more embedded in social context and in valued cooperation (Gudykunst et al., 1996; Kanai and Yukawa, 2017), compared to a more “independent” self-construal of Westerners, including a greater individualization focus of uniqueness, and less informed by the social context and a focus on contextual cues. This could render Asians less attentive to their internal bodily states relative to external cues stemming from the external, social world (see Ma-Kellams, 2014). This means that for Japanese the individual self is not the central object of focus or primary unit of analysis compared to the surrounding context, which in turn would make bodily changes to a lesser degree the focus of attention.

Although it has been highlighted that East Asians tend to show greater cultural emphasis on body processes and find bodily features more salient in everyday life as well as report higher levels of somatic “sensibility” than people of Western cultures (Ma-Kellams, 2014), it has also been demonstrated that interoceptive perception accuracy, as measure by heartbeat perception tasks is lower in East Asians (Ma-Kellams et al., 2012). This has been suggested to be related to contextual attention bias, i.e., that Asians tend to disproportionately focus on external contextual entities outside of themselves, both in terms of other individuals (interdependent self) (Markus and Kitayama, 1991), as well as other factors in their environment, as has been demonstrated in experimental paradigms (Kitayama et al., 2003). This may hamper the ability to accurately infer bodily changes. The FFMQ- AWA scale aims at assessing actions in the moment, i.e. a process that is also connected to environmental and social context. Thus, cultural differences in context-relatedness of self-construal, might explain why “Noticing” (awareness of all body sensations: comfortable, uncomfortable or neutral) and “Emotional Awareness” (attribution of physical sensations with physiological manifestations of emotions) are not positively related to the FFMQ-AWA (“Acting with Awareness”) in the Japanese sample in comparison to US samples of the original MAIA.

Going beyond convergent validity issues of MAIA-J and FFMQ, and focusing on divergent validity, our results showed that all MAIA-J scales demonstrated negative or non-significant correlations with DERS subscales (−0.24 to −0.32) and STAI trait anxiety (−0.23 to −0.49), suggesting that emotion regulation difficulties and trait anxiety are also negatively associated with bodily awareness measures of MAIA-J. This is according to the original MAIA results (Mehling et al., 2012) and underscores MAIA-J construct validity.

However, correlations with STAI trait anxiety were slightly lower than in the English MAIA study. Additionally, there were no significant correlations between MAIA-J scales “Noticing” as well as “Emotional Awareness” and STAI trait anxiety. This is different to the significant and negative correlations of all original MAIA scales with STAI trait anxiety in the US samples (Mehling et al., 2012). This finding may support our suggestion that either “Noticing” and “Emotional Awareness” in Japanese are more “overlapping” constructs and might be less clearly differentiated from anxiety-related and/or negative evaluations compared to Western cultures, or that the difference is due to Westerners in the original English validation were more experienced in mind-body practices.

Interestingly, PCS scales were also negatively or non-significantly correlated to MAIA-J scales (−0.34 to 0.09), which is similar to the original MAIA study, but with the slight exception of the PCS scale “Magnification.” This subscale was slightly positively correlated to the MAIA-J scale “Noticing.” In the original MAIA study with US samples of mind-body experienced individuals, all MAIA scales were clearly negatively correlated with PCS subscales (Mehling et al., 2012). In this context, it has to be stated again that MAIA-J “Noticing” scale demonstrated a different item structure, including items with emotion-related content. PCS assesses catastrophizing in response to pain sensations, and the “Magnification” subscale describes “the concern that the pain will get worse or have negative outcome.” In combination with our suggestions regarding results for MAIA-J “Noticing” scale and FFMQ AWA (“Acting with Awareness”) subscale, the STAI trait findings may also underscore the idea of different emotional evaluation of bodily sensations in Japanese as compared to Western samples (see also Ma-Kellams, 2014).

In summary, discussion of the validation of MAIA-J as has been investigated in this study requires consideration of two relevant aspects. Firstly, as discussed above, but without going into further detail for the complex topic of culturally bound epistemologies and cross-cultural research, our findings of the MAIA-J factor structure may reflect existing cross-cultural differences, that are expected to affect multidimensional interoceptive awareness as assessed by self-report.

On a more general note, differences in the factor structure of the MAIA-J can be viewed in the light of findings demonstrating that people refer to internal bodily sensations when describing their emotional experiences (Nummenmaa et al., 2014), and that this categorical association is likely reinforced by cultural consensus, translating perceived interoceptive responses into a social language that supports emotional understanding of self and others (Critchley and Garfinkel, 2017). Based on more indirect evidence, existent literature suggests that East-Asians may show greater cultural emphasis on body processes than people of Western cultures (Ma-Kellams, 2014). In contrast, experimental data suggest that East-Asian show a weaker ability to accurately perceive bodily changes as well as a greater misattribution of the causes of their bodily changes (Ma-Kellams et al., 2012). This divergence has been explained, again, by greater context-relatedness and interdependency of self-perception in Japanese culture. In accordance with this, interesting findings by Maister and Tsakiris (2014) highlighted that integrated experience of interoceptive and exteroceptive signals differs between East Asian and Western cultures. The authors showed that an exteroceptive self-focus (viewing one's face) does not improve interoceptive accuracy in East Asians in contrast to Western participants. Instead, for East Asian individuals, the external appearance of the self may activate higher-level, social aspects of self-identity, reflecting the importance of the sociocultural construct of “face” in East Asian cultures. This finding underscores that basic cultural differences in self-construal indeed prominently influence the sense of the bodily self and are of importance when assessing self-reported dimensions of bodily awareness.

Secondly, another factor that has to be recognized regarding the factor structure of the MAIA-J are differences in sample characteristics compared to the original MAIA version. The original English MAIA and its 8-factor structure has been developed and validated in US-American individuals trained in mind-body strategies, whereas our findings were assessed in healthy young Japanese adults without mind-body training experience. It has been demonstrated that training of interoceptive awareness practices positively influences MAIA dimensions, especially the regulatory aspects of interoceptive awareness, that is how the body is used for self-regulation (Bornemann et al., 2015). Therefore, beyond discussed cultural differences, differences in sample characteristics of interoceptive awareness training experience may play a role in the slightly different MAIA-J factor structure, especially regarding its regulatory dimensions. This may show up in the weak factor loadings of the Self-Regulation dimension of the MAIA-J that was deleted in our Japanese sample. Nevertheless, the dimension Attention Regulation that has also been demonstrated to be positively affected by interoceptive trainings in non-trained samples (Bornemann et al., 2015) is not different in the MAIA-J, and showed factor loading results comparable to the original MAIA.

We cannot exclude that sample differences in training experiences may have affected the results. However, there is also evidence showing that the original 8-factor structure of the MAIA does not fundamentally depend on pre-selected sample characteristics of mind-body trained vs non-trained samples. On the one hand, the conceptual model of the MAIA with its eight dimensions has been confirmed in primary care patients with past or current low back pain that had no prior experience with body-mind therapies (Mehling et al., 2013). On the other hand, “Western” (European) MAIA validation studies, such as the Italian MAIA (Calì et al., 2015) and the German MAIA (Bornemann et al., 2015), not only included mind-body trained samples, but either both, trained and non-trained, or only non-trained healthy young adults, mostly university students, which is comparable to our sample. Interestingly, both Italian and German MAIA versions found an equivalent 8-factor structure comparable to the original MAIA. Additionally, the Korean MAIA validation results of exploratory factor analyses (Wan-Suk et al., 2016) demonstrated a different factor structure including different item-factor loadings with 32 items and 6 altered MAIA dimensions. The Korean MAIA version has been reported to show sufficient reliability (internal consistency of 0.94) and construct validity. Regarding sample characteristics, the Korean MAIA study included healthy young adults with both low and high experience in trainings related to interoceptive awareness.

Taken these lines of evidence together, it is not sufficient to suggest that differences in MAIA-J and original MAIA factor structure are primarily based on sample differences. Therefore, interpretation of the MAIA-J results suggests to reflect also cultural and conceptual (language related) alterations of interoceptive awareness as assessed by self-report.

Indeed, an interesting and relevant next step for future studies will be to investigate the Japanese MAIA in different Japanese samples, such as those with low and high experience in mind-body trainings, in order to test its reliability and validity in different populations, and to further disentangle the influences of effects of pre-existing interoceptive awareness experience and existent cultural differences in more detail.

## Conclusion

Our study offers the Japanese translation and validation of the MAIA to be used for further research. The findings of our study demonstrate that MAIA-J represents a slightly different factor structure compared to the original English MAIA, likely due to cultural differences. The study results support acceptable reliability and validity of the MAIA-J in the Japanese sample, warranting its use for future studies with Japanese populations and for cross-cultural studies.

## Ethics statement

The studies were carried out in accordance with the recommendation of the Guideline by the Ethics Committee of the Medical Faculty of the University of Tuebingen (Germany) with written informed consent from all subjects. All subjects gave written informed consent in accordance with the Declaration of Helsinki. The protocol was approved by the Ethics Committee of the Medical Faculty of the University of Tuebingen (Germany).

## Author contributions

BH: conceived and designed the study, mentoring, contributed materials and analysis tools, data analyses, writing the paper; MS: data acquisition, data analyses, co-writing; WM: co-writing, collaboration, contributed materials; MH: co-writing, contributed materials.

### Conflict of interest statement

The authors declare that the research was conducted in the absence of any commercial or financial relationships that could be construed as a potential conflict of interest.

## APPENDIX

### Japanese translation of original MAIA items

1.
2.
3.
4.
5.
6.
7.
8.
9.
10.
11.
12.
13.
14.
15.
16.
17.
18.
19.
20.
21.
22.
23.
24.
25.
26.
27.
28.
29.
30.
31.
32.

### MAIA (MAIA-J)

1. (1.)
2. (2.)
3. (4.)
4. (5.)
5. (6.)
6. (7.)
7. (11.)
8. (12.)
9. (13.)
10. (14.)
11. (15.)
12. (16.)
13. (17.)
14. (18.)
15. (19.)
16. (20.)
17. (21.)
18. (22.)
19. (26.)
20. (27.)
21. (28.)
22. (29.)
23. (30.)
24. (31.)
25. (32.)

(numbers of original MAIA items in parenthesis)
